# Classification Performance of Machine Learning Methods for Identifying Resistance, Resilience, and Susceptibility to *Haemonchus contortus* Infections in Sheep

**DOI:** 10.3390/ani13030374

**Published:** 2023-01-21

**Authors:** Luara A. Freitas, Rodrigo P. Savegnago, Anderson A. C. Alves, Ricardo L. D. Costa, Danisio P. Munari, Nedenia B. Stafuzza, Guilherme J. M. Rosa, Claudia C. P. Paz

**Affiliations:** 1 Department of Genetics, University of Sao Paulo, Ribeirão Preto 14049-900, SP, Brazil; 2 Department of Animal and Dairy Sciences, University of Wisconsin, Madison, WI 53706, USA; 3 Department of Animal Science, Michigan State University, East Lansing, MI 48864, USA; 4 São Paulo Agency of Agribusiness and Technology, Animal Science Institute, Nova Odessa 13380-011, SP, Brazil; 5 School of Agricultural and Veterinary Sciences, São Paulo State University, Jaboticabal 14884-900, SP, Brazil; 6 Sustainable Livestock Research Center, Animal Science Institute, São José do Rio Preto 15130-000, SP, Brazil

**Keywords:** multinomial logistic regression, *Ovis aries*, precision, sensitivity

## Abstract

**Simple Summary:**

Infection by gastrointestinal nematodes is a major sanitary issue in sheep production. Therefore, improvements in the animal’s health are important to reduce losses and improve animal welfare. Thus, this study investigated the feasibility of using easy-to-measure phenotypic traits to predict sheep resistant, resilient, and susceptible to gastrointestinal nematodes, compared the classification performance of different methods, and evaluated the applicability of the best classification model on each farm. The results revealed the multinomial logistic regression and linear discriminant analysis models presented the best classification performances for the susceptible and resistant animals. The results suggest that the use of readily available records and easily measurable traits may contribute to the identification of susceptible animals, supporting management decisions at the farm level and potentially reducing the economic losses due to parasitic infection. The animals identified as resistant can also be incorporated as selection candidates into breeding programs for the genetic improvement of sheep populations.

**Abstract:**

This study investigated the feasibility of using easy-to-measure phenotypic traits to predict sheep resistant, resilient, and susceptible to gastrointestinal nematodes, compared the classification performance of multinomial logistic regression (MLR), linear discriminant analysis (LDA), random forest (RF), and artificial neural network (ANN) methods, and evaluated the applicability of the best classification model on each farm. The database comprised 3654 records of 1250 Santa Inês sheep from 6 farms. The animals were classified into resistant (2605 records), resilient (939 records), and susceptible (110 records) according to fecal egg count and packed cell volume. A random oversampling method was performed to balance the dataset. The classification methods were fitted using the information of age class, the month of record, farm, sex, Famacha© degree, body weight, and body condition score as predictors, and the resistance, resilience, and susceptibility to gastrointestinal nematodes as the target classes to be predicted considering data from all farms randomly. An additional leave-one-farm-out cross-validation technique was used to assess prediction quality across farms. The MLR and LDA models presented good performances in predicting susceptible and resistant animals. The results suggest that the use of readily available records and easily measurable traits may provide useful information for supporting management decisions at the farm level.

## 1. Introduction

Infection by gastrointestinal nematodes is a great sanitary concern for the sheep meat industry, causing, directly and indirectly, economic impacts due to the compromised health conditions of infected animals [1]. The negative impacts of gastrointestinal nematodes include subclinical weight loss and lethal pathologies such as anemia, severe protein loss, reduced feed intake due to anorexia, and in cases of massive infection, high mortality rates [2,3,4]. The most pathogenic blood-feeding parasitic nematode in sheep is *Haemonchus contortus*, which is responsible for causing anemia and great economic losses [5]. The indiscriminate use of anthelmintics to reduce infections by gastrointestinal nematodes resulted in serious problems of parasite resistance to anthelmintics and increased production costs [6]. The combined annual cost of the helminth infections in 18 European and neighbor countries was estimated at €1.8 billion, with 81% percent of this cost due to lost production and 19% attributed to treatment costs [7].

An alternative to reduce the problems associated with anthelmintics is the detection of animals according to their response to parasitic infection and consequently the breeding of sheep for resistance to nematodes, i.e., sheep with the immunological capacity to control parasitic infection [8,9]. The Santa Inês is a breed of hair sheep derived from crosses of Morada Nova, Bergamasca, and the native coarse-wooled Crioula, which present a higher resistance to gastrointestinal nematodes than Suffolk and Ile de France sheep [10].

Sheep can be classified into three classes according to their response to parasitic infection: resistant, resilient, and susceptible [11]. Resistant animals are less prone to be infected because their infection response acts to avoid the establishment of the parasite, without damage to production [12]. Resilience is related to the animal’s ability to keep production performance in the face of a disease challenge [12,13]. Susceptible animals are those that host the parasites and have a high degree of anemia followed by impaired production. Currently, infection level can be quantified by fecal egg count and packed cell volume [13,14,15,16]. However, these parameters depend on blood and feces collection, which are difficult to perform and require laboratory resources that increase costs for farmers. Cheaper, non-invasive, and non-laborious alternatives to classify the animals’ infections response must be explored in order to reduce productions costs. 

Machine learning model (ML) theory represents a branch of artificial intelligence that combines statistics, computer science, and data mining principles aiming to find and learn inherent patterns and to classify (or predict) interest outcomes. MLs have been used for classification in many areas. Most of them are generalizations or extensions of linear models to make them more flexible to accommodate nonlinear relationships between variables, dealing with collinearity and high dimensional data [17,18]. These classification algorithms have been increasingly used in different tasks in the livestock industry, such as for identifying the estrus occurrence in dairy cows [19], detection of different pathologies in animal necropsy reports [20], and classification of dairy cattle breeds [21], with the most suitable method depending on the problem studied.

Although there are several studies using those models for classification, to the best of our knowledge, the potential of using ML to classify resistance, resilience, and susceptibility to gastrointestinal nematode infections in sheep populations has not been investigated yet. Hence, the objectives of this study were (1) to investigate the feasibility of using easy-to-measure phenotypic traits to predict sheep resistant, resilient, and susceptible to gastrointestinal nematodes, (2) to compare the classification performance of different methods (multinomial logistic regression—MLR, linear discriminant analysis—LDA, random forest—RF, and artificial neural network—ANN), and (3) evaluate the applicability of the best classification model on each farm.

## 2. Materials and Methods

### 2.1. Data Set

The dataset comprised 3654 phenotypic samples of 1250 Santa Inês sheep recorded in two periods: from 2013 to 2014 and from 2018 to 2020. The animals came from six farms: Cravinhos (latitude: 21°20′25″ S; longitude: 47°43′46″ W), Jardinopolis (latitude: 21°01′04″ S; longitude: 47°45′50″ W), Nova Odessa (latitude: 22°46′39″ S, longitude: 47°17′45″ W), Pontal (latitude: 21°01′21″ S; longitude: 48°02′14″ W), Serrana (latitude: 21°12′41″ S; longitude: 47°35′44″ W), and Ventania (latitude: 24°14′45″ S; longitude: 50°14′34″ W). The experimental procedures were conducted following the recommendations of the Institutional Animal Care and Use Committee of the Animal Science Institute, Nova Odessa, São Paulo, Brazil (protocol code CEUA Nº. 267-18, 3 October 2018).

The feces were collected directly from the rectal ampoule and individually subjected to fecal egg count using the modified McMaster technique of Gordon and Whitlock [22]. A pool of feces samples was separated for the preparation of larvae culture to establish the genera of nematodes prevalent in the herd [23]. The results showed that the main genus of gastrointestinal nematode found during the study was *Haemonchus* (63%) followed by *Trichostrongylus* (24%), *Cooperia* (7%), and *Oesophagostomum* (6%) [24].

Blood samples were collected in 5 mL vacutainer tubes containing EDTAK3 by puncture of the jugular vein. The packed cell volume was measured by the microhematocrit centrifugation technique [25]. Resistant animals (2605 records) had fecal egg counts equal or less than 1000 eggs/g and packed cell volume equal or higher than 22%. The resilient animals (939 records) had fecal egg counts over 1000 eggs/g and packed cell volume higher or equal to 22%, and the susceptible animals (110 records) had packed cell volume less than 22%.

Body weight was measured with an electronic scale. Body condition score was obtained by visual inspection and by palpation of the dorsal lumbar region of the spine, constituting scores from 1 to 5 and their intermediates (1.5, 2.5, 3.5, and 4.5), which represent a (1) cachectic animal; (2) thin animal; (3) moderate animal; (4) fat animal; and (5) obese animal [26,27]. The missing observations for body weight and body condition score were imputed considering the animal’s most recent valid measure.

The Famacha© diagnosis was performed by trained technicians using the Famacha© card, which compares the different shades of the ocular conjunctiva on a five-point scale corresponding to the colors robust red (non-anemic—1), red/pink (non-anemic—2), pink (mildly anemic—3), pink/white (anemic—4), and white (severely anemic—5) [28].

### 2.2. Sampling Technique 

The different classes (resistant, resilient, and susceptible) were not equally represented in our data sets (Table S1). As it is well known that prediction models can have poor prediction performance with highly unbalanced datasets, the method used to overcome the unbalanced dataset issue was the random over-sampling method, which randomly replicates instances in the minority class. The random oversampling method was performed through the R software using the caret package (R Core Team, 2018).

### 2.3. Classification Models

Multinomial logistic regression (MLR), linear discriminant analysis (LDA), random forest (RF), and artificial neural network (ANN) methods were used to classify the animals according to their response to the parasitic infection. The hyperparameter values were set to defaults for the MLR, LDA, and RF models. Different architectures were tested for the ANN model, and the architecture with the best performance in our training set was that with a single layer and 7 hidden neurons, 200 epochs, the sigmoidal activation function in the hidden layer, and the softmax function in the output layer. The analyses were performed with the R software (R Core Team, 2018), using the randomForest package for random forest analyses [29], the MASS package for linear discriminant analyses, and the nnet package for artificial neural networks and multinomial logistic regression analyses [30].

The response variable was the animal class (resistant, resilient, and susceptible, coded as 0, 1, 2, respectively). The exploratory variables were body weight (kg), body condition score (scores 1 to 5 and their intermediates), farm, sex (3224 female records and 430 male records), age class (194 records in class 1: 1 to 6 months of age, 588 records in class 2: 6 to 18 months of age, and 2872 records in class 3: over 18 months of age), month of record and Famacha© degree (from 1 to 5; [28]).

### 2.4. Classification Performance Metrics

For comparison purposes, the whole dataset was randomly split into training and testing sets, which contained 80% and 20% from the original number of observations, respectively. Classification metrics were calculated using 5-fold cross-validation with 20 replicates. Precision and sensitivity (%) were used for comparing the classification methods for each class. The classification metrics were calculated as follows:

$$\mathrm{Precision}\;=\frac{\mathrm{TP}}{\left(\mathrm{TP}+\mathrm{FP}\right)}\times 100\%$$

$$\mathrm{Sensitivity}\;=\frac{\mathrm{TP}}{\left(\mathrm{TP}+\mathrm{FN}\right)}\times 100\%$$

in which TP = true positive rate, FP = false positive rate, and FN = false negative rate.

Sensitivity was used to select the best model for classifying susceptible animals. Sensitivity is important when it is concerned with identifying positive outcomes (parasitized animals) and the cost of a false positive is low (animals classified as susceptible when they are not), as long as the model identifies as many actual positives as possible. Precision was used to select the best models to classify resistant and resilient animals. Precision looks at the ratio of true positives to the predicted positives. This metric is most often used when there is a high cost for having false positives (in this case, classification of resistant or resilient animals when they indeed are not).

In addition, the accuracy and AUC of the models studied were calculated. The AUC corresponds to the area under the ROC curve, whose maximum value is 1.0, indicating that the model is 100% sensitive and 100% specific [31]. The AUC was obtained using the auc function with multcap object of the R program (R Development Core Team, 2018).

### 2.5. Extrapolating Classification across Farms 

After finding the best classification model, a leave-one-farm-out cross-validation technique was performed by excluding all the data from a target farm in the training set and using it in the testing set. We did this for exploring the classification performance of the models to predict animal classes in a specific farm that was not used to build the model. Therefore, out of the six farms in the dataset, five farms were included in the model training, whereas the data from one farm was held out as testing set in each run of the cross-validation, and the process was then repeated for each farm.

## 3. Results

### 3.1. Prediction Models

The different classes were not equally represented in the data, with the percentage of animals classified as resistant, resilient, and susceptible being 71.3%, 25.7%, and 3.0%, respectively. After balancing the classes by the random over-sampling method, we tested four different models to classify sheep according to their level of gastrointestinal parasite infection. 

**Figure 1:**
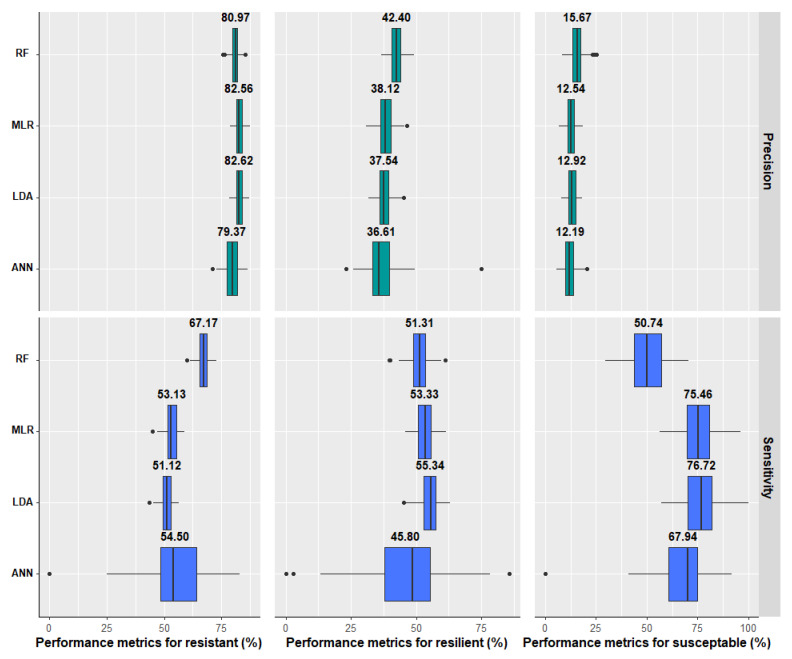
Precision and sensitivity for the classification of parasitic infection response (resistant, resilient, and susceptible) in sheep according to different methods (random forest—RF, multinomial logistic regression—MLR, linear discriminant analysis—LDA, and artificial neural network—ANN) trained with easy-to-measure explanatory variables. Performance metrics were computed using a 5-fold cross-validation with 20 random replicates. The left and right of the box represent first and third quartiles, respectively; the vertical line denotes the median; the value above the box is the mean; the whiskers correspond to 1.5 × interquartile distance; and the dark dots are outliers.

Among the four models tested, both MLR and LDA provided the best performances in predicting susceptible animals, as indicated by the high values of sensitivity (75.5% and 76.7%, respectively) compared to the RF and ANN models (Figure 1). The poorest performance for this infection response class was achieved with the RF, which identified only 50.7% of the susceptible animals (Figure 1). The precision of the MLR was 82.6% for resistant animals, whereas this metric ranged from 36.6 to 42.4% for resilient animals, suggesting poor performance for this specific class (Figure 1).

The mean accuracy ranged between 59% (ANN) and 63% (MLR and RF) for resistant animals, between 58% (ANN) and 63% (RF) for resilient animals, and between 71% (RF) and 80% (MLR and LDA) for susceptible animals (Table 1). In addition to the performance metrics (sensitivity and precision) and accuracy, the AUC was also used to assess the performance of the tested models (Table 1). The best performance (AUC = 0.78) was obtained for the MLR and LDA.

### 3.2. Classification Performance of Leave-One-Farm-Out Cross-Validation Technique

**Figure 2:**
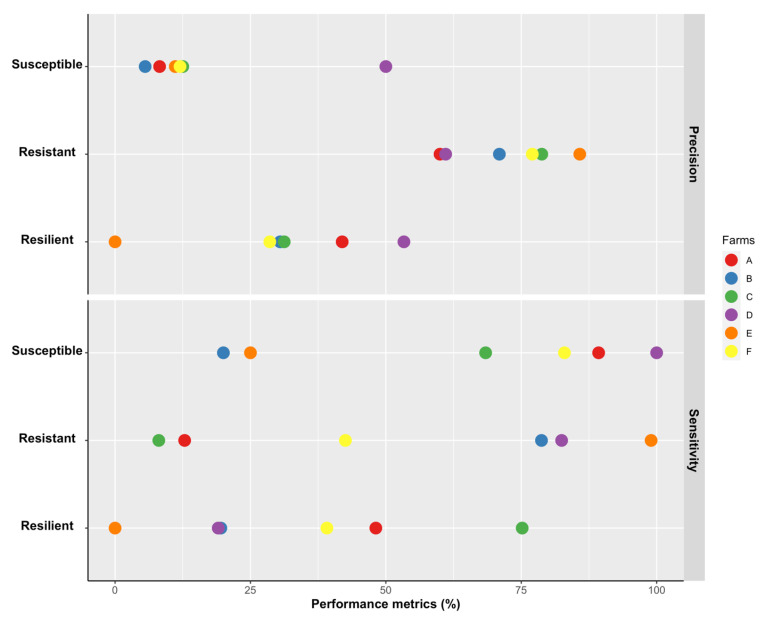
Precision and sensitivity of multinomial logistic regression model using sex, age class, month of record, body weight, body condition score, and Famacha© degree as explanatory variables and response classes of the parasitic infection (resistant, resilient, and susceptible) as the response variable for each farm. Performance metrics were calculated leave-one-farm-out cross-validation technique, alternating the data from one specific farm as the testing set and the remaining as training sets.

According to the results, the MLR was one of the most suitable methods for classifying resistant and susceptible animals (Figure 1). We further investigated the performance of this model across farms by alternating the data from one specific farm as the testing set and the remaining as training set. The sensitivity for the MLR model considering different farms ranged from 20 to 100% for the susceptible class, presenting better results in farms A (89.3%), D (100.0%), and F (83.0%) (Figure 2). While the precision for resistance ranged from 60.0 to 85.8% with the best results for farm B (71.0%), farm C (78.8%), farm E (85.8%), and farm F (77.0%) (Figure 2), the identification of resilient animals showed low precision in all farms, ranging from 0 (Farm E) to 53.3% (Farm D).

## 4. Discussion

In this study, sensitivity was used to select the best model to classify susceptible animals. This metric indicates how good a given model is at predicting the sick animals (susceptible animals) when the cost of a false positive is low (animals classified as susceptible when they are not) as long as the model identifies as many parasitized animals as possible. The cost of a high rate of false positives becomes low considering that the farmer treats all animals identified as susceptible. As treatment of all animals in the herd is a common management in many sheep farms, and false positives in this case are not a big issue in the production system.

Precision was used to select the best models to classify resistant and resilient animals. This metric is most often used when there is a high cost for having false positives (in this case, the classification of susceptible animals as resistant or resilient). A high precision rate for the classification of resistant animals reduces the risk of not treating real susceptible animals that are incorrectly identified as resistant or resilient, contributing to increased contamination of pastures and a higher cost with treatments.

Poor precision values were observed for the minority class (susceptible animals), and this was mostly because this metric is sensitive to the class distribution. Additionally, by performing a simple random oversampling for a minority class with too few datapoints, all observations and explanatory variables tend to appear repeatedly in the training data. It happens because this method just samples with replacement from the original data, which can contribute to an overfitting during the training process.

Therefore, considering the criteria used to compare the classification performance across models, the most suitable model combines higher sensitivity for susceptible animals and the highest precision for resistant and resilient animals. This specific criterion was chosen by considering the balance among productivity, cost reduction, and treating the parasitized animals in the herd in order to reduce the contamination of pasture and other animals while increasing the profitability of the production system and making it more sustainable. It was found that among the four models tested, the MLR model presented one of the best classification performances for the susceptible and resistant animals. The precision for resilient animals was low in all models, so it was not possible to correctly identify the best model for this class. Still, the precision average for the investigated models was higher than the expected random guess threshold for resilient class (roughly 26%), suggesting that the models are capturing relevant information from the data.

The MLR and LDA models presented higher AUC (0.78). According to Hosmer and Lemeshow [32], an AUC of 0.7 to 0.8 is considered acceptable. Thus, considering a model that combines higher sensitivity for susceptible animals and higher precision for resistant and resilient animals and presents an acceptable AUC, MLR and LDA can be classified as good for predicting classes of parasitic infection.

Excluding animals or part of the records for the same animal from the training data set are strategies generally employed for validating the model predictive ability [33,34]. Shetty et al. [34] reported that when multiple observations related to the same cow are only partially omitted from the training set, the model predictions tend to be more optimistic. Dorea et al. [33] investigated this topic hypothesizing that even when excluding an animal from the training data set, conditions related to external factors such as weather, diet, season, management, and others would inflate the prediction quality of other animals in the same circumstances. These authors concluded that inflation in the prediction ability occurred when animals from the same trial were kept in both the training and validation datasets. In the present study, we investigated this issue by hypothesizing that conditions related to the farm would inflate the prediction quality assessment. Clearly, inflation in the classification performance occurred when sheep from all farms were split randomly and the repeated measures of the same animal were present in both training and validation datasets. Our approach to validate the models by excluding an entire farm from the training dataset is closer to reality because the developed models are supposed to be used to predict new datasets coming from farms with different external factors, such as diets, weather, and management practices.

The applicability of the present study in sheep farming was validated by analyzing the classification performance by farm. This approach revealed that the MLR model was able to predict with good performance both classes (resistant and susceptible) in two farms (C and F) and at least one of the classes in four farms (susceptible class in Farms A and D or resistant class in Farms B and E). The proposed approach could help attenuating the negative impacts related to infections caused by gastrointestinal nematodes, contributing to design deworming strategies that take into account the risk of an animal being contaminated, consequently reducing the costs with anthelmintic administration and laboratory analyses based on blood or fecal samples.

The identification of resistant and susceptible animals predicted through easily measurable, non-invasive, and cost-reduced variables can be used to support animal/herd management decisions in which animals identified as resistant to gastrointestinal nematodes may be incorporated as selection candidates in breeding programs, whereas animals classified as susceptible can receive adequate treatment.

Easy-to-measure variables were used in the models due to their association with gastrointestinal parasite infection. Several studies reported that gastrointestinal nematode infections had a negative effect on meat production and that performance of sheep infected with nematodes was 85% of the performance in uninfected individuals for weight gain [1]. According to Cornelius et al. [35], body condition score showed promise as a selection index under commercial farming conditions. One survey showed that body condition score was the best criterion to detect infected sheep, with only 1.1% of false negatives [36]. Another extremely useful tool for identifying parasitized animals through the diagnosis of anemia is the Famacha© method [28]. Sheep infected with blood-sucking *H. contortus* may show anemia, eosinophilia, and hypoproteinemia, resulting in pale mucous membranes and submandibular edema [5], which seems to be the case in the present study considering the higher prevalence of *H. contortus* (63%) in the herd. Additionally, procedures requiring blood sampling and laboratory services of individuals is more expensive in terms of time and resources, which makes them less attractive for monitoring purposes. Therefore, cheaper alternatives must be studied to reduce the cost of production. 

The use of models with easy-to-measure phenotypic traits for identification with high sensitivity of the susceptible animals allows treatment of only those animals instead of the whole herd. This brings many advantages to the farmer, such as reducing the cost with laboratory resources and with the use of anthelminthics, considered an expensive drug and, in most cases, only partially effective [37]. Furthermore, overuse and frequent use of anthelmintics have resulted in substantial and widespread problems with anthelmintic resistance in nematode populations [38,39]. Another advantage is the reduction in the number of agricultural residues deposited in meat and soil, increasing the environmental sustainability of the farm [40]. Therefore, there are large economic and environmental gains by increasing the control of the main parasitic diseases in sheep.

Overall, the use of prediction algorithms in sheep farming is still incipient. There are no reports in the literature on the use of machine learning models for classifying resistance, resilience, and susceptibility to gastrointestinal nematodes in sheep. This reinforces the importance of the present work, which certainly opens the way for further studies and future applicability at the field level.

## 5. Conclusions

Multinomial logistic regression and linear discriminant analysis achieved the best performances for classifying susceptible and resistant animals. The results suggest that the use of readily available records and easy-to-measure variables such as body weight, body condition score, farm, sex, age class, record month, and Famacha© degree may contribute to the identification of susceptible animals, supporting management decisions at the farm level and potentially reducing the economic losses due to parasitic infection with higher prevalence of *H. contortus* parasites. The animals identified as resistant can also be incorporated as selection candidates into breeding programs for genetic improvement of sheep populations.

## Figures and Tables

**Table 1:** Mean and standard deviation of the performance metrics of the multinomial logistic regression (MLR), linear discriminant analysis (LDA), random forest (RF), and artificial neural network (ANN) methods using sex, age class, month of record, body weight, body condition score, and Famacha© degree as explanatory variables and response classes of the parasitic infection (resistant, resilient, and susceptible) as the response variable.

Performance Metrics		Model ^2^			
		MLR	LDA	RF	ANN
AUC ^1^		0.78 (0.02)	0.78 (0.02)	0.77 (0.02)	0.73 (0.04)
Accuracy	Resistant	0.63 (0.02)	0.62 (0.02)	0.63 (0.02)	0.59 (0.03)
	Resilient	0.62 (0.02)	0.62 (0.02)	0.63 (0.02)	0.58(0.03)
	Susceptible	0.80 (0.04)	0.80 (0.04)	0.71 (0.05)	0.76 (0.05)

^1^ AUC = area under the ROC curve. ^2^ Multinomial logistic regression (MLR), linear discriminant analysis (LDA), random forest (RF), and artificial neural network (ANN) methods. The value of AUC for a multiple class response by averaging pairwise comparisons is the mean of 5 repeats of cross-validation with 20 random replicates. The value of accuracy is the mean of 5 repeats of cross-validation with 20 random replicates.

## Data Availability

The data presented in this study are available on request from the corresponding author. The data are not publicly available due to privacy or ethical restrictions, and the data that support the findings of this study are available in the supplementary material of this article.

## Supplementary Materials

The following supporting information can be downloaded at: https://www.mdpi.com/article/10.3390/ani13030374/s1, Table S1: Number of records for each farm and each class of parasitic infection (resistant, resilient, and susceptible).
