# Supplementary material for: Classification Performance of Machine Learning Methods for Identifying Resistance, Resilience, and Susceptibility to Haemonchus contortus Infections in Sheep
Source: Animals (Basel). 2023 Jan 21;13(3):374. doi: 10.3390/ani13030374 (PMC9913374; doi:10.3390/ani13030374)
Supplement: Supplementary file 1 [file animals-13-00374-s001.zip › animals-2135117-supplementary.pdf]

Supplementary Materials

Table S1. Number of records for each farm and each class of the parasitic infection (resistant, resilient and susceptible).

Class of the parasitic infection	Farm					
	A	B	C	D	E	F
Resistant	397	301	322	57	684	844
Resilient	243	128	145	42	110	271
Susceptible	28	5	19	7	4	47
